# CMV retinitis and subsequent acute cystoid macular oedema after treatment with vitreous ganciclovir injection: a case report

**DOI:** 10.1186/s12886-022-02500-0

**Published:** 2022-06-28

**Authors:** Mengyun Liu, Hengqian He, Qinkang Lu, Juntao Zhang

**Affiliations:** Department of Ophthalmology, The Affiliated People’s Hospital of Ningbo University, The Eye Hospital of Wenzhou Medical University (Ningbo Branch), Ningbo, 315040 People’s Republic of China

**Keywords:** CMV retinitis, Acute cystoid macular oedema, Ganciclovir, Case report

## Abstract

**Background:**

To report a very rare acute cystoid macular oedema following ganciclovir injection in patients receiving allogeneic haematopoietic stem cell transplantation.

**Case presentation:**

A 44-year-old male patient experienced vision loss in his left eye eight months after allogeneic stem cell transplantation. Ophthalmologic examination showed posterior retinopathy with retinal haemorrhage, a yellow necrotic border, and a vascular white sheath involved in the superior temporal retina but not the posterior pole. Cytomegalovirus DNA results in both plasma and ocular fluid were positive. All tests combined with the patient’s medical history suggested that his ocular disease was cytomegalovirus retinitis. Consequently, he received a weekly ganciclovir vitreous injection. The disease was visibly controlled, and the fundus condition improved after the first three treatments. However, the patient had severe vision loss in his left eye and acute cystic oedema in the macula, while the original lesion was stable two hours after the fourth treatment. The macular oedema subsided significantly on the first day. Over the next week, daily OCT findings indicated that the patient's macular oedema gradually subsided and resolved completely by the second week, and his left eye vision partially improved.

**Conclusion:**

Macular oedema may occur in patients with cytomegalovirus retinitis, but it rarely occurs during treatment. In this case, the patient's macular oedema appeared and resolved quickly. Macular oedema in patients with cytomegalovirus retinitis receiving vitreous cavity injections of ganciclovir needs to be further studied and discussed.

## Background

Cytomegalovirus (CMV) is a common enveloped double-stranded DNA virus that belongs to the herpes viral family [1]. Cytomegalovirus retinitis is associated with immunocompromised hosts. It occurs in neonates, in bone-marrow and solid-organ-transplant recipients and in patients with acquired immunodeficiency syndrome (AIDS) infected with human immunodeficiency virus (HIV). The presence of CMV retinitis is correlated with the level of immunosuppression [2].

CMV retinitis, a type of cytomegalovirus disease, is strongly associated with mortality in allogeneic haematopoietic stem cell transplant (HCT) recipients [3–5].

CMV retinitis clinically presents as full-thickness retinal necrosis, oedema, or exudative detachment [6, 7]. Some studies have reported that methotrexate combined with oral valganciclovir alleviates CMV-induced cystoid macular oedema with a better prognosis [2]. Currently, typical treatments for CMV retinitis include intravenous and intravitreal injections of ganciclovir and foscarnet, intravenous injections of cidofovir, and oral administration of valganciclovir [8]. Intravitreal injection with the administration of various pharmacological agents to treat different eye diseases can improve intraocular drug concentrations and reduce systemic side effects. However, intravitreal injection of the drug may still cause retinitis, intraocular inflammation, rhegmatogenous retinal detachment, increased intraocular pressure, and fundus haemorrhage [9].

Furthermore, some rare complications, such as retinal vein and arterial occlusion [10, 11], haemorrhagic macular infarction [12], anterior ischaemic optic neuropathy [13, 14], or ocular ischaemic syndrome [15], may also be present. Most of these complications occur after anti-VEGF injections. Ganciclovir is a popular therapy for preventing and relieving CMV retinitis in HCT recipients [16, 17].

There are few reports of side effects caused by intraocular ganciclovir injections. In this case report, we report a haematopoietic stem cell transplantation recipient who presented with acute cystoid macular oedema following intraocular ganciclovir injection.

## Case presentation

A 44-year-old Chinese man with no history of hypertension or diabetes who had received allogeneic stem cell transplantation for 8 months was admitted to our hospital for a progressive decrease in visual acuity in his left eye. The patient had diffuse large B-cell lymphoma and had received an allogeneic haematopoietic stem cell transplant after chemotherapy. His blood tested positive for CMV during the treatment period, but no ocular symptoms were present.

**Fig. 1:**
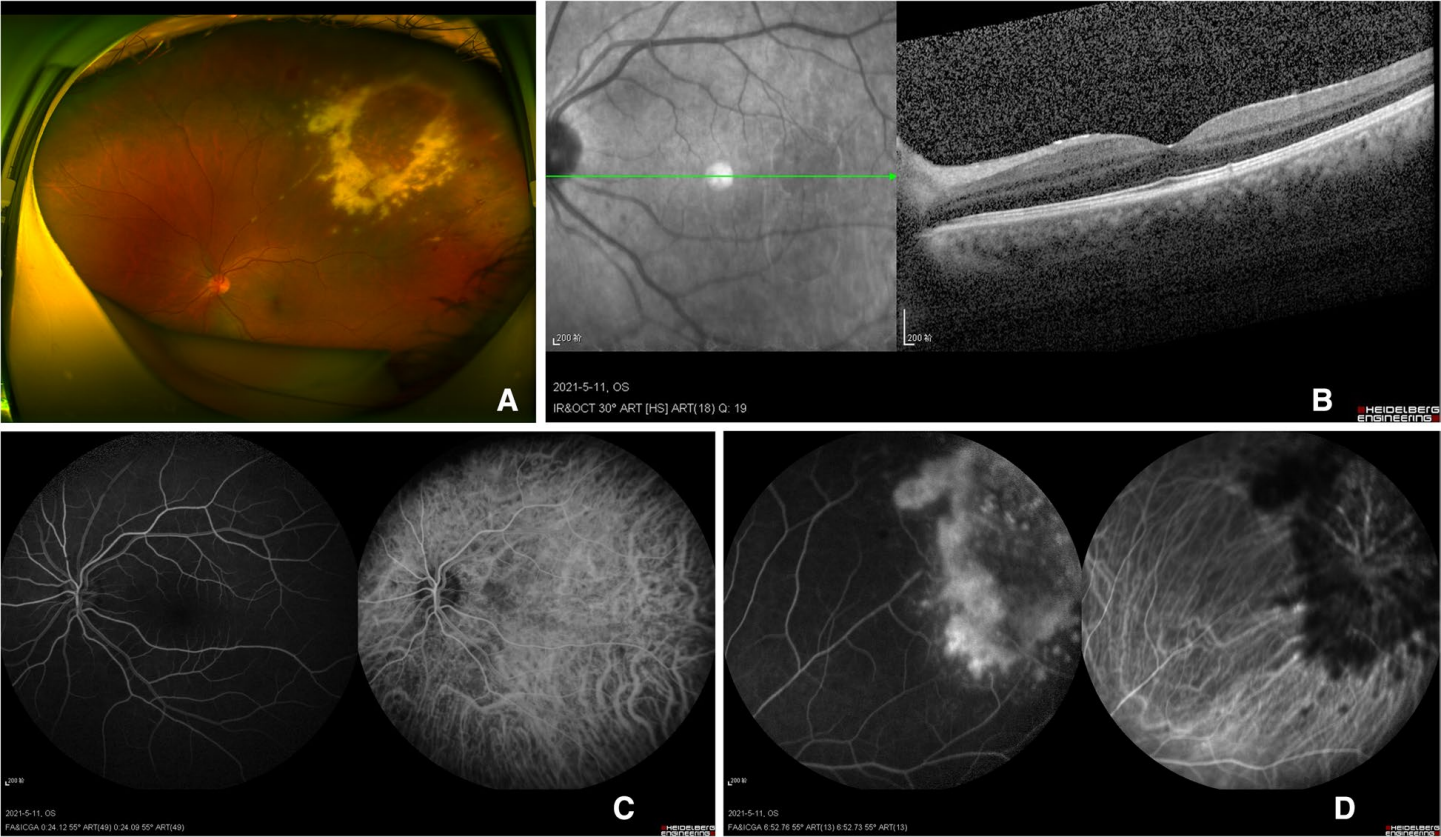
**A** Fundus photography showed haemorrhage and vascular white sheaths in the superior temporal region of the left eye. **B**, **C** OCT (**B**), FFA, and ICGA (**C**) showed a normal appearance of the posterior pole of the left retina. **D** Fluorescence leakage and obscured fluorescence in the superior temporal retina were detected by FFA and ICGA

However, the patient developed ocular symptoms at 8 months after receiving the transplant. Hence, he received his first ocular examination in the ophthalmology department. We determined that the patient had an uncorrected visual acuity (UCVA) of 20/500 and a best-corrected visual acuity (BCVA) of 20/40 in both eyes using the Snellen visual acuity chart. Slit-lamp examination showed that there was no significant abnormality in his anterior segment, except for increased lens density. However, endoscopy revealed blebs, sheets of necrosis, and a white vascular sheath anteriorly in the patient's left eye. Based on the patient’s previous history, we further refined the fundus photography, OCT, and FFA + ICGA (Fig. 1). He had no significant abnormalities in the macular area, according to OCT. At the same time, his left eye had degenerated in the superior temporal area because of haemorrhage, and the white vascular sheath was not close to the macula according to fundus photography. The extent of the lesion involved the superior macular temporal vessels. It crossed the equator and approached the ora serrata. We found a fluorescence leak in the lesional region by the FFA + ICGA exam. No significant abnormalities were observed in his right eye. We suspected the presence of CMV retinitis in the patient’s left eye based on the medical history and ophthalmologic examination.

**Fig. 2:**
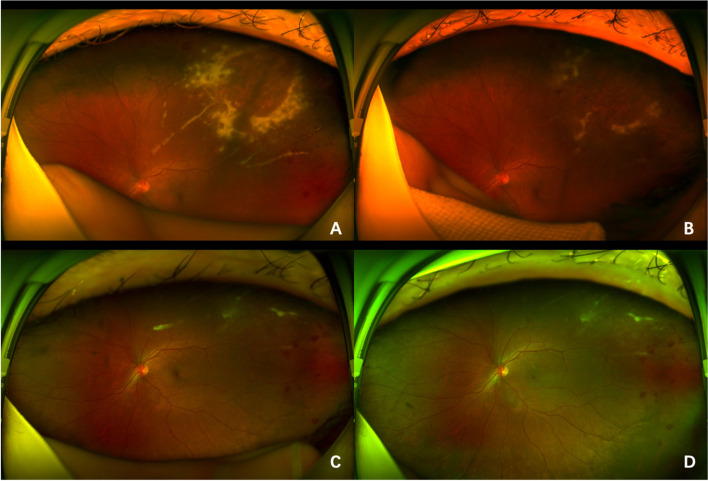
**A**, **B**, **C**, and **D** represent photographs of the lesions after the first, second, third and fourth intravitreal injections, respectively

Consequently, we extracted the patient’s anterior chamber aqueous fluid for viral nucleic acid and antibody testing. It showed a nucleic acid copy number of 1.55 × 10^4^ and an antibody quantity of 16.25 U/ml per ml of CMV, which was well above the normal reference range. Meanwhile, nucleic acid tests for other viruses, including HSV, EBV, VZV, and HHV-6, were negative. These results made the diagnosis of CMV retinitis in the patient's left eye fairly clear. On May 18, May 25, June 1 and June 8, 2021, the patient received intravitreal injections of 4 mg ganciclovir. The extent of the fundus lesions and vascular white sheaths was reduced after the first three treatments (Fig. 2).

**Fig. 3:**
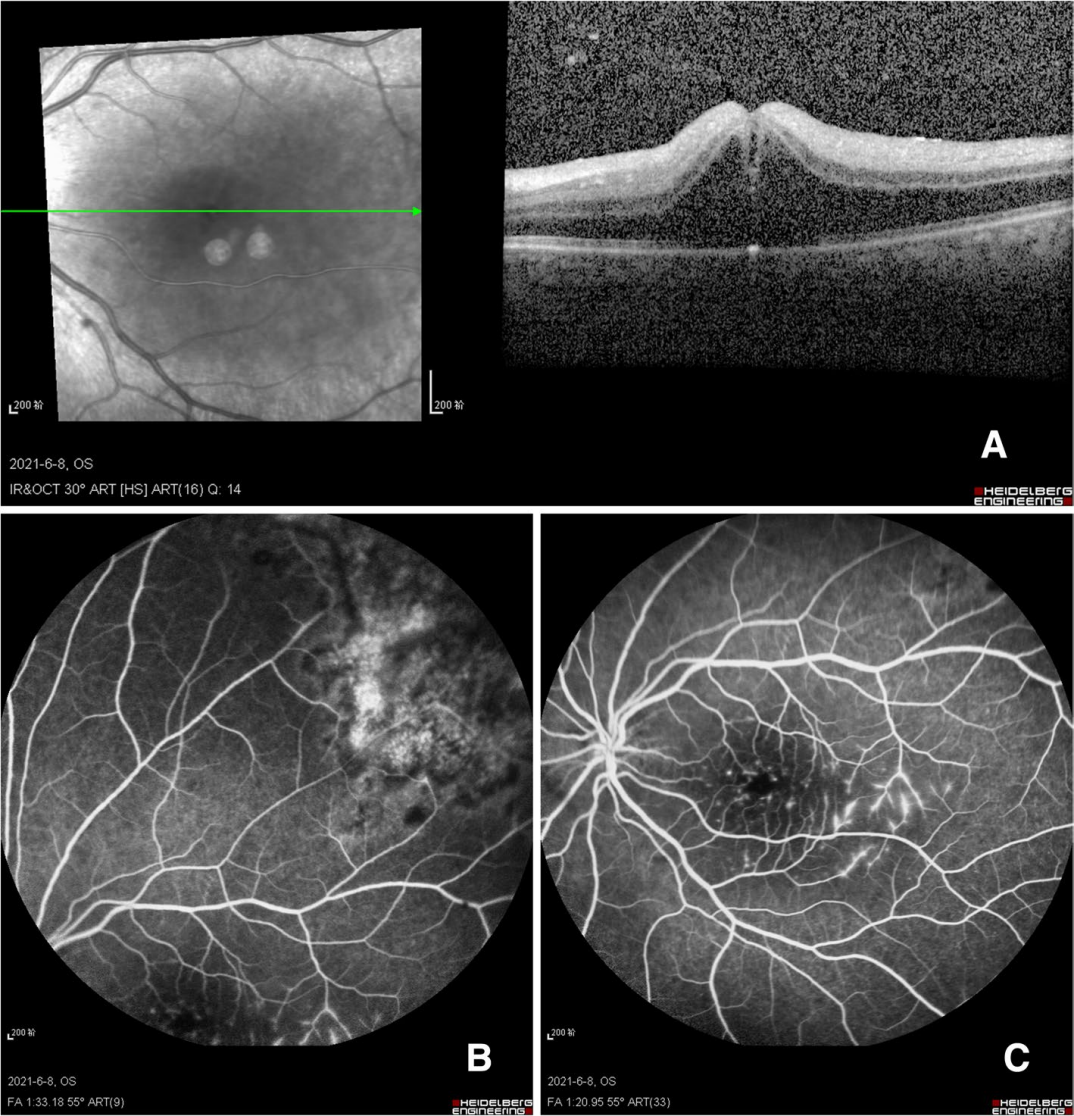
**A** OCT examination showed cystoid macular oedema after the fourth injection. **B** A fluorescence leak in the macula was revealed by angiography. **C** The original lesion had no obvious fluorescence leakage

**Fig. 4:**
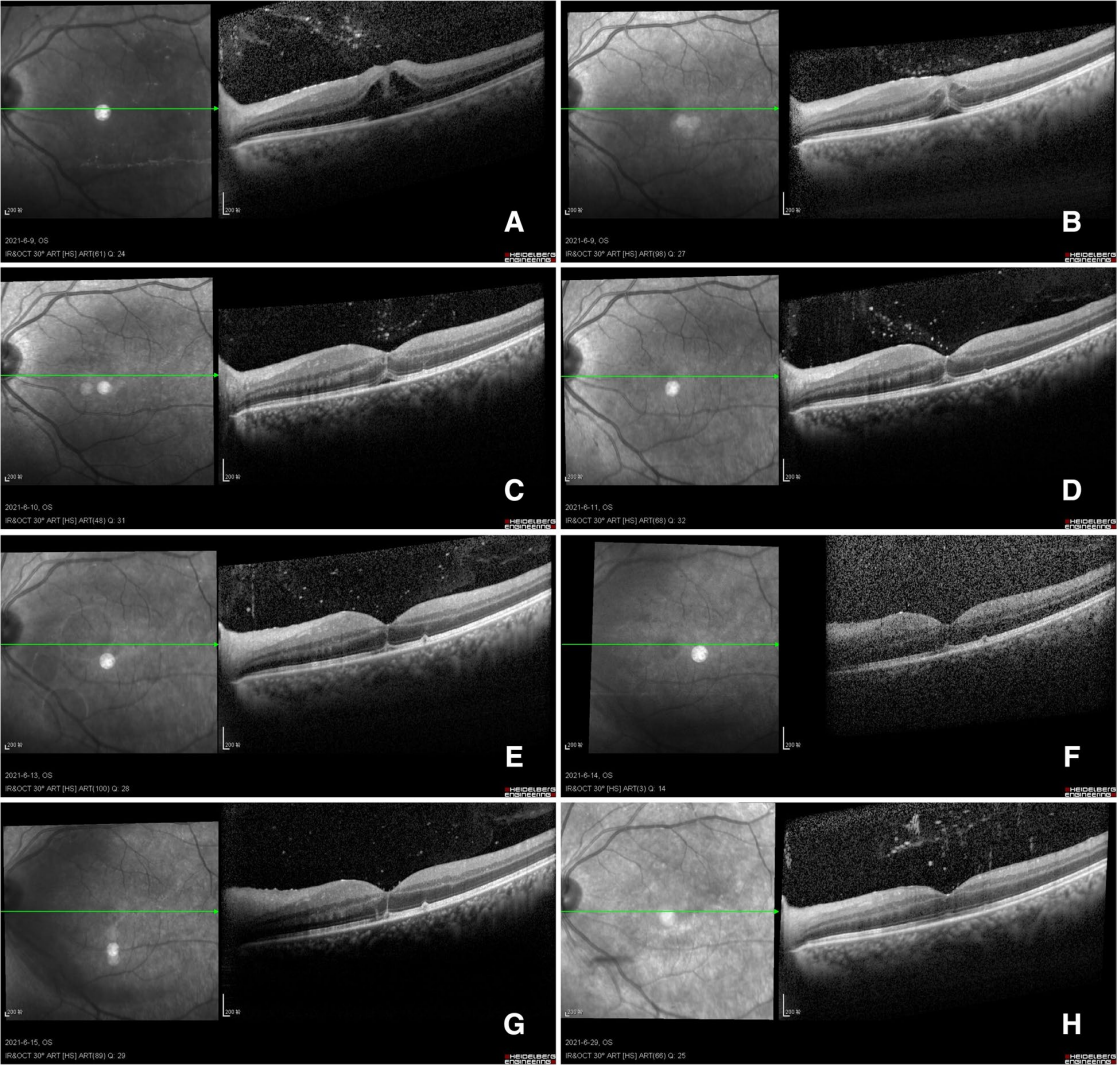
These images represent OCT images of the macula during the first week and the second week after the fourth injection. The macular oedema basically subsided within one week, and there was no obvious morphological change in the macula in the second week. **A** OCT image taken on the second morning. **B** Image taken on the second afternoon. **C**, **D** Images from the third and fourth days. **E**, **F**, **G** Images from day six to day eight. **H** Images after three weeks

Nonetheless, the patient complained of a sharp vision loss in his left eye for two hours after the fourth therapy. The BCVA of his left eye suddenly dropped to 20/1000, and we immediately performed OCT, FFA, and fundus photography on his left eye (Fig. 3). The OCT results suggested cystoid macular oedema in the left eye, which seemed to have accumulated only in the outer five layers of the retina and appeared as a vertical dense signal strip below the fovea. The FFA finding revealed a fluorescence leak in the macula and paramacular (temporal to the fovea). However, the result of fundus photography showed no indication of the progression of the original lesion. These findings suggested that paramacular vasculitis might be the aetiology of cystoid macular oedema. However, according to the OCT image, the place where the vasculitis occurred was not the most obvious place for leakage, and there was no clear evidence for a CMV aetiology. Next, we repeated the anterior puncture and aspirated the patient's intraocular fluid. The sample of the ocular fluid was negative for the CMV nucleic acid assay. We then monitored the patient and found a marked improvement in cystoid macular oedema by that afternoon. The cystoid macular oedema had largely disappeared with minimal sub-RPE fluid by the next day. During the following days, the macular oedema continually improved, and the superficial peeling of the RPE layer disappeared after four days. Since then, the cystoid macular oedema has not occurred again. The patient's BCVA ranged between 20/400 and 20/100 over this period. The patient had his final ophthalmic follow-up on March 22, 2022. His BCVA in the left eye was 20/400, and OCT showed no significant macular abnormalities (Fig. 4).

## Discussion and conclusion

CMV retinitis usually occurs in patients with AIDS, with immunosuppression, and with previous intravitreal glucocorticoid implantation [8, 14, 15]. CMV retinitis develops on average 8 months after bone marrow transplantation. Weekly intravitreal injections of 4 mg ganciclovir are the standard treatment for cytomegalovirus retinitis. Cystoid macular oedema is one of the clinical manifestations of CMV retinitis [8]. CMV retinitis patients with cystoid macular oedema have poorer vision, longer virus duration, and poor prognosis [18]. Cystoid macular oedema usually develops before the injection of ganciclovir. Interestingly, in the present case, cystoid macular oedema occurred rapidly after intravitreal ganciclovir injection, and the majority of the oedema dispersed on the same day, showing a rapid appearance and quick disappearance. It did not recur during the following months, and the patient’s vision remained stable. It has been previously documented that non-AIDS patients who develop CMV retinitis can also develop cystoid macular oedema when immune medication is reduced or antivirals are added [19]. This outcome may be associated with ganciclovir resistance [19]. However, the present patient's past medical history showed that he had received no immunosuppressive therapy or systemic antiviral medication in the 2 months prior to the fourth injection, and there was no evidence of ganciclovir resistance. This finding suggests a possible relationship between cystoid macular oedema and the vitreous injection of ganciclovir.

The cause of macular oedema remains unclear. In previous reports, RVO and RAO occurred after intravitreal injection, and RVO also caused cystoid macular oedema [20]. Nevertheless, RVO and RAO were denied due to several exams showing no delayed vascular filling and obstruction findings. However, a few studies have reported retinal toxicity following intravitreal ganciclovir injections. This patient did not develop macular oedema until the fourth injection. Therefore, it is unlikely that drug toxicity caused the macular oedema.

Macular cystoid oedema occurred in the outer five layers of the retina, as repeatedly observed in the OCT images. The blood to these five layers was supplied by the choroid. Accordingly, it is reasonable to speculate that macular cystoid oedema may be induced by transient choroidal ischaemia. Despite this being a sensible conjecture for this macular oedema, it still fails to explain the vertical hyperreflective signal bars beneath the macular area in the OCT images. Having reviewed much of the literature, we still do not have a more plausible explanation. The treatment of CMV retinitis in non-AIDS patients usually comprises a combination of corticosteroids and NSAIDs [19]. In this case, the patient was placed under close observation after the development of macular oedema. Fortunately, his cystic macular oedema disappeared quickly, and his vision was restored to some extent.

Macular oedema may occur in patients with cytomegalovirus retinitis, but it rarely occurs during treatment. Macular oedema in patients with cytomegalovirus retinitis receiving vitreous cavity injections of ganciclovir needs to be further studied and discussed.

In the case of a similar and longer persisting situation occurring again, intraocular glucocorticoid therapy combined with NSAID eye drops can be considered while ensuring negative intraocular CMV results.

## Data Availability

Supporting data for the results of this study are available from the authors upon request.

## Abbreviations

CMV: Cytomegalovirus
AIDS: Acquired immunodeficiency syndrome
HIV: Human immunodeficiency virus
HCT: Haematopoietic stem cell transplant
UCVA: Uncorrected visual acuity
BCVA: Best-corrected visual acuity
OCT: Optical coherence tomography
FFA: Fundus fluorescein angiography
ICGA: Indocyanine green angiography
HSV: Herpes simplex virus
EBV: Epstein Barr virus
VZV: Varicella-Zoster virus
HHV-6: Human herpesvirus 6
RPE: Retinal pigment epithelium
RVO: Retinal vein occlusion
RAO: Retinal artery occlusion
